# The Effects of Yoga on Fall-Related Physical Functions for Older Women: A Systematic Review of Randomized Controlled Trials

**DOI:** 10.3390/healthcare13020124

**Published:** 2025-01-09

**Authors:** Tzu-Chun Huang, Ching Li, Ching-Yu Hsieh

**Affiliations:** 1 Graduate Institute of Sport, Leisure and Hospitality Management, National Taiwan Normal University, Taipei City 106308, Taiwan; 2 MSc Business Analytics, University College London, London WC1E 6BT, UK

**Keywords:** balance, gait, lower-limb strength, multicomponent exercise

## Abstract

**Introduction:** The evidence showed that the risk of falls was higher in women, and yoga was considered an effective rehabilitation method for preventing falls. However, there had been no previous attempts to synthesize the evidence specifically for the use of yoga in preventing falls among older women. **Objectives**: This systematic review aimed to strengthen the existing body of evidence by focusing exclusively on the impact of yoga in improving fall-related physical functions among older women. **Methods**: A systematic review was conducted following the PRISMA guidelines. The protocol was developed in advance of the study and registered on PROSPERO (Registration number: CRD42024506550). **Results**: The effects of yoga on balance, gait, and lower-limb strength were inconsistent. It showed that yoga programs designed to prevent falls in older women might not demonstrate the same effectiveness as those identified in previous systematic reviews for the general older adult population. **Conclusions**: This systematic review is the first to exclusively explore the impact of yoga on fall-related physical functions in older women. However, this review did not directly observe a definitive effect of yoga on fall prevention in older women. Future studies should delve deeper into identifying appropriate yoga postures and determining the optimal dose required to enhance physical function and prevent falls.

## 1. Introduction

Taiwan transitioned into an aged society in 2018 and is anticipated to progress into a super-aged society by 2025 [1], raising significant concerns about age-related diseases and health issues. Among these health concerns, falls and their associated injuries significantly increase with age, making falls a common health issue among the elderly. According to a survey conducted in Taiwan involving 3280 individuals aged 65 and above, 15.5% of the respondents reported experiencing a fall in the past year [2]. It is noteworthy that women had a higher risk of falling than men [3], with the prevalence of falls reported as 29.1% among older women and 23.5% among older men [4]. Previous studies speculated that there are various intrinsic factors that make women more prone to falls than men, such as history of osteoporotic fracture after menopause, worse physical performances [5], and lower knee muscle strength [6]. Therefore, the development of fall prevention programs tailored to the unique needs of older women is becoming increasingly important.

Exercise has been proven to be an effective strategy for fall prevention among older adults [7]. Studies have indicated that poor physical function is related to a high risk of falls [8,9]. Specifically, Song et al. [10] observed that older women in Taiwan who were at risk of falling exhibited issues with gait, balance problems, and weakened leg muscles. Consequently, exercise training aimed at enhancing functional fitness, specifically balance and mobility, has been considered an effective approach to reduce the risk of falls [11,12]. Yoga was an exercise found to be predominantly favored by a female population [13,14]. As one of the complementary health practices, yoga has been recommended for the elderly to enhance both physical function and mental well-being if there are no clinical contraindications [15,16]. Therefore, the discussion regarding whether yoga is an effective strategy for preventing falls has also increased [17,18].

Two systematic review and meta-analysis studies have assessed the effects of yoga on functional fitness factors related to the risk of falling for elderly, showing small to moderate enhancements in balance, mobility, and lower-limb strength associated with yoga [19,20]. Shin [21] not only confirmed the positive impact of yoga on improving physical function but also found that a nine to twelve-week yoga program had a significant effect on older adults. These empirical studies demonstrated the favorable effects of yoga on balance and mobility, offering preliminary evidence for yoga as a potential intervention to prevent falls in older adults. While evidence has shown that the risk of falls is higher in women [22], yoga, a popular and well-accepted exercise among women [13,14], is considered effective rehabilitation for the prevention of falls among elderly [17]. There have been no previous attempts to synthesize the evidence specifically for the use of yoga in preventing falls among older women. Youkhana et al. [19] and Sivaramakrishnan et al. [20] included studies with both male and female subjects to evaluate the effects of yoga on fall prevention, which made it challenging to distinctly examine the impact of yoga on fall prevention in older women. According to global guidelines for falls in older adults [23], fall risk factors associated with fitness function encompass balance, gait, and lower-limb strength. Therefore, this systematic review aimed to strengthen the existing body of evidence by focusing exclusively on the impact of yoga in improving fall-related physical functions among older women. Specifically, we sought to address the following research questions:

(1) What is the effect of yoga exercise on balance in females aged 60 and older?
(2) What is the effect of yoga exercise on gait in females aged 60 and older?
(3) What is the effect of yoga exercise on lower-limb strength in female aged 60 and older?

## 2. Materials and Methods

### 2.1. Search Strategy

A systematic review was conducted following the Preferred Reporting Items for Systematic Reviews and Meta-Analyses (PRISMA) guidelines. The protocol was developed in advance of the study and registered on PROSPERO (Registration number: CRD42024506550). In total, four electronic databases were searched for literature, including EBSCO, ProQuest, Scopus, and PudMed, in January 2025. Search terms were (elder OR older OR ag* OR senior) AND (wom*n OR female) AND (yoga) AND (fall OR physical OR balanc* OR mobility OR strength OR gait), in reference to previous review articles [19,20]. A detailed list of search terms and a search strategy based on Boolean Logic is presented in the Appendix A. The reference list of relevant systemic reviews was also hand-searched [24]. Article type was restricted to randomized controlled trial (RCTs) in all four databases, with inclusion criteria for both English and Chinese articles, without imposing limitations on the publication years.

### 2.2. Screening and Selection Criteria

Screening was conducted in three stages using reference management software (EndNote 20). Initially, one author (TCH) performed a preliminary screening of titles and abstracts, during which duplicates and obviously irrelevant studies were excluded. Subsequently, two researchers (TCH, CYH) independently screened the titles and abstracts of all studies, categorizing them as “Yes” (meeting eligibility criteria), “No” (not meeting eligibility criteria), or “Maybe” (uncertain and requiring further examination). Finally, two researchers (TCH, CYH) conducted a detailed examination of the full texts of studies, categorized as “Yes” and “Maybe”. In cases of disagreement, a third researcher (CL) resolved the discrepancies.

Using the population, intervention, control, and outcome (PICO) approach, the inclusion and exclusion criteria for studies were as follows: (i) Participants: older women were defined as females with a mean age of 60 years and above, with no restrictions on the basis of specific disease or condition. Our study exclusively included clinical trials targeting older women and excluded studies where the data included both men and women to ensure a focused and meaningful analysis tailored to this specific demographic; (ii) Intervention: studies using yoga as an intervention, without restrictions on the specific type of yoga, such as hatha yoga, sauna yoga, chair yoga, or other forms, were included. Studies in which yoga was specified as control condition or combining with other exercise were excluded; (iii) Outcome: Only studies reporting one of the physical function outcomes related to falls risk factor on functional fitness [23], including balance, gait, and lower-limb strength, were included; (iv) Study type: studies with a randomized (including cluster randomized) controlled study design published in English were included.

### 2.3. Data Extraction

Two authors (TCH, CYH) independently assessed the participant characteristics, sample sizes, yoga program, content of experimental and control groups, measurement protocols, as well as the outcomes in the chosen articles. This study exclusively incorporated randomized controlled trials (RCTs) and evaluated outcomes to determine significant differences between the experimental and control groups. The level of significance was set at *p* < 0.05. Additionally, to report the strength of the yoga intervention, the effect size (Cohen’s d) was calculated and categorized as follows: trivial (*d* < 0.2), small (0.2 < *d* < 0.6), moderate (0.6 < *d* < 1.2), large (1.2 < *d* < 2.0), very large (2.0 < *d* < 4.0), and nearly perfect (*d* > 4.0) [25]. In cases of disagreement regarding an article, it was subjected to further review by another author (CL), and discussions were held until a consensus was reached.

### 2.4. Quality Assessment

The quality assessment of the included studies was conducted by evaluating their PEDro scale scores, which were obtained from the Physiotherapy Evidence Database (https://pedro.org.au/) (accessed on 1 January 2025). The PEDro scale serves as an evaluative tool for appraising trial methodologies, encompassing 11 distinct items [26]. The initial item pertains to external validity, while the subsequent 10 items contribute to computing the overall score, ranging between 0 and 10. Its primary objective is to aid users in discerning trials with robust internal validity (items 2–9) and those providing enough data for meaningful interpretation of their findings (items 10–11). It is worth noting that, in the context of yoga-based interventions, the highest attainable PEDro score is often restricted to 8 out of 10, as the blinding of the treating instructor and participants presents inherent challenges [19].

## 3. Results

### 3.1. Flow of Studies Through the Review

**Figure 1:**
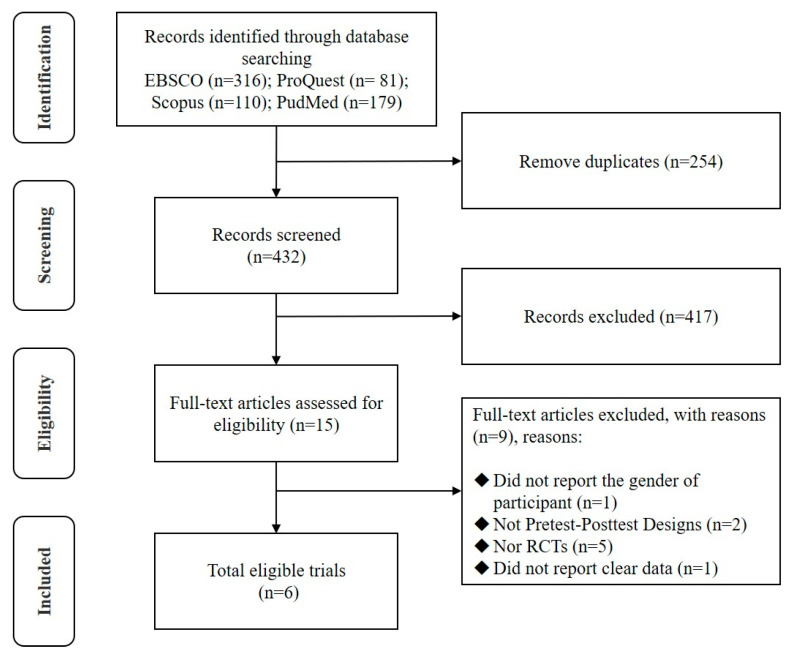
Flow of studies through the review.

From the initial data searches, a total of 732 records were identified, of which 717 were excluded due to duplication or failure to meet the criteria. Fifteen full-text articles were assessed for eligibility [27,28,29,30,31,32,33,34,35,36,37,38,39,40,41]; eight articles were excluded because they did not employ pretest–posttest designs [37,39] or were not randomized controlled trials [28,29,30,31,35,41]. An additional full-text was excluded because it did not report complete results [40]. Six RCTs reporting on yoga for participants with fall risk factors, encompassing 169 participants, were finally included in the analysis (Figure 1) [27,32,33,34,36,38].

### 3.2. Study and Participant Characteristics (Table 1)

Six RCTs with a total of 170 participants were included in the analysis. The sample sizes ranged from 18 to 38, with a median of 28 participants per trial. Three RCTs were conducted in the United States [33,34,38], while the other three originated from Thailand [27], Canada [36], and Portugal [32]. The participants were recruited based on various health conditions: overweight or obesity [27], knee osteoarthritis [36,38], chronic pain [33], and no specified clinical condition [32,34]. The mean age of the participants ranged from 62 to 83.7 years, with a median age of 69 years.

### 3.3. Intervention Characteristics and the Types of Yoga

The length of interventions ranged from four to 28 weeks (median: 10 weeks). Frequency varied from one to three sessions per week, with a median of two sessions per week. Session duration ranged from 50 to 60 min (median: 60 min) per session. Overall, participants received between 480 and 3500 min of contact time with the instructor (median: 1440 min). In all studies, the control group received an inactive intervention, avoiding additional exercise beyond standard usual care. One study provided additional insights by comparing yoga with strengthening exercises for knee osteoarthritis [36]. Another RCT conducted outcome measurements at two different time points to assess the influence of intervention duration [27]. All RCTs measured adverse events associated with the yoga intervention; however, no adverse events were reported.

Six RCTs encompassed yoga interventions (Table 2) that comprised various elements, such as yoga postures, breathing techniques, strengthening exercises, meditation, and relaxation. Among these studies, the specific type of yoga was unspecified in one case [34].

### 3.4. Quality

The mean PEDro score among the selected studies was 6.75. All six trials implemented randomization and concealed allocation. Specifically, four studies attained a score of 7 out of 10 on the PEDro scale, while the remaining three achieved a score of 6 out of 10. Comprehensive details of the PEDro scores can be found in Table 1. The details of the quality assessment of each study are shown in Table 3.

### 3.5. Outcome Measures

Balance was assessed in three out of six studies using different methods: the functional reach test [27], one-leg-stand test [34], and the balance test of the short physical performance battery [38].

Gait measures were assessed in five out of six studies using various tests, including the eight-foot up and go test [27,32,33,36,38], the six-minute walk test [27,36], a 40 m walk [36], and the two-minute step test [32,33]. Notably, the eight-foot up and go test was the only measurement used consistently across all these studies for assessing gait.

Five out of 6 studies assessed lower limb strength using the 30 s chair stand test [27,33,36] and the five times sit-to-stand test [34,38].

### 3.6. Outcomes

#### 3.6.1. Effects of Yoga on Balance

The effects of yoga on balance were inconsistent. After four to eight weeks of yoga intervention, only one out of three studies reported a significant improvement in balance, demonstrate a nearly perfect clinical relevance [27]. In this RCT, an 8-week Thai yoga program focusing on older women with overweight or obesity demonstrated notable balance improvement. However, the other studies [34,38] found no significant differences between the yoga and control groups. It is important to note that Widjaja et al. [27] did not assess balance improvement after 4 weeks of intervention but observed it after 8 weeks of intervention.

#### 3.6.2. Effects of Yoga on Gait

The effects of yoga on gait were inconsistent. Although two studies showed significant improvements in gait, inconsistent results were observed in a 28-week chair-based yoga intervention [32]. While there was a significant difference in gait assessed by the 2 min step test, this was not reflected in the results obtained from the eight-foot up and go test [32]. Therefore, only one study confirmed a significant and consistent improvement in gait after an eight-week Thai yoga program for older women with overweight or obesity, demonstrating nearly perfect clinical relevance [27]. However, three out of five RCTs did not find a significant difference in gait compared to either the inactive control group or the strengthening exercise for knee osteoarthritis [33,36,38].

#### 3.6.3. Effects of Yoga on Lower-Limb Strength

The effects of yoga on lower-limb strength were inconsistent. Two out of five RCTs found a significant improvement in lower-limb strength after 8 weeks of Thai yoga and Hatha yoga for older women with overweight or knee osteoarthritis, demonstrating very large to nearly perfect clinical relevance [27,38]. In the contract, the other three RCTs showed a non-significant difference in lower-limb strength [33,34,36].

## 4. Discussion

To the best of our knowledge, this is the first systematic review of RCTs exclusively examining the impact of yoga on improving fall-related physical functions among older women. Previous research has established improving fall-related physical functions as an effective fall prevention strategy [11,12]. Therefore, this review provides valuable insights into the feasibility of using yoga as a fall prevention strategy for older women.

The main finding of this study suggests that the effect of yoga on fall prevention for older women is still unclear. It shows that yoga programs designed to prevent falls in older women may not demonstrate the same effectiveness as those identified in previous systematic reviews for the general older adult population [19,20,21]. This implied that the effect of yoga on fall prevention in older women might exhibit different influential factors. Previous studies have indicated that women generally experience more frequent and severe issues related to sarcopenia, functional capacity, frailty, and disability [42]. These physiological mechanisms might influence the effectiveness of yoga on fall prevention in older women. Emerging evidence supports the idea that considering sex differences is crucial when devising strategies for fall prevention [43,44]. This finding holds significant implications for intervention designers and public health practitioners. When designing effective yoga programs for older women to prevent falls, it is crucial to consider their specific physiological needs.

The notable finding of this study suggests that yoga programs incorporating lower-limb-strength-training poses, such as the mountain pose, warrior poses, tree pose, and similar postures, can enhance the effectiveness of fall prevention in older women. Although previous systematic reviews have argued that the effectiveness of yoga interventions does not necessarily depend on the specific style of yoga [45], the yoga poses in an intervention should align with the intended therapeutic goal. In this review, several included studies had diverse objectives, such as managing knee osteoarthritis [38], reducing pain [33], and enhancing psychological well-being [32,34], rather than focusing specifically on improving functional fitness to prevent falls. This variation in study goals likely contributed to the inconsistent findings. Future research should prioritize yoga types and postures that are known to strengthen the lower limbs and improve functional fitness in fall prevention programs for older women.

Furthermore, the effectiveness of yoga interventions may also be influenced by the frequency and duration of practice. The findings suggest that if the goal of a yoga program is to enhance fall-related physical functions, the intervention should last for at least eight weeks. Although this review included yoga postures known to enhance functional fitness, there were variations in the frequency and duration of yoga practice across the studies. This heterogeneity in frequency and duration of yoga practice poses challenges in comparing findings across studies and limits our understanding of how yoga impacts physical well-being. In this review, only one included study had outcome assessments at different time points [27]. The results indicated that the improvement in balance was not assessed after four weeks of intervention but was observed after eight weeks. This finding is consistent with a prior study that demonstrated a significant effect on older adults’ physical function after a nine to twelve-week yoga program [21]. Future studies should investigate the optimal duration of yoga practice to enhance physical function in the elderly population.

Although the direct effect of yoga on fall prevention was not observed in this review, the potential contribution of yoga to fall prevention may play a supportive role. Particularly, it may serve to enhance the exercise motivation of older women, given that yoga remains a popular and widely accepted form of exercise among women. Previous studies have indicated that exercise training should focus on aspects such as balance, strength, agility, and varying intensities to reduce injurious falls and the number of injured fallers [46,47]. Emerging evidence indicated that a multicomponent exercise program has a better effect on fall prevention [23]. The guideline of the World Health Organization also recommends that older adults should engage in diverse multicomponent physical activities at least three days a week [48]. It helps preventing falls and fall-related injuries, as well as reducing declines in bone health and functional ability. Therefore, yoga can be used as an adjunct intervention to enhance exercise motivation for older women while incorporating strength, aerobic, balance, gait, and flexibility training as a multicomponent exercise fall prevention program.

This review possesses both strengths and limitations. Its strengths lie in the inclusion of only RCTs and focusing exclusively on the population of older women, indicating a high-quality evidence base regarding the effect of yoga in improving fall-related physical functions in this demographic. However, the primary limitation of this review is the heterogeneity among the included studies, particularly regarding clinical conditions such as overweight or knee osteoarthritis, which increases the difficulty of drawing a definitive conclusion. This variability also significantly impacts the feasibility and interpretability of conducting a meta-analysis. Additionally, differences in yoga intervention protocols further contribute to the challenges in drawing consistent conclusions. To better evaluate the effectiveness of yoga in preventing falls among older women, future RCTs should standardize participant profiles and study designs while including larger sample sizes and populations with similar clinical conditions to provide more robust evidence. Finally, determining the most effective method for assessing the risk of bias in trials of yoga interventions remains uncertain. While we opted to utilize the PEDro scale for measuring bias, the Cochrane risk of bias tool has also been suggested and applied. Further discussion is needed to determine the appropriate method for assessing the risk of bias.

## 5. Conclusions

This systematic review has examined the impact of yoga on improving fall-related physical functions in older women. While the effectiveness of yoga in preventing falls among older women remains uncertain, the findings indicate that incorporating lower-limb-strength-training poses, such as the mountain pose, warrior poses, and tree pose, can enhance the effectiveness of yoga programs for fall prevention. Additionally, interventions should last for at least eight weeks to achieve improvements in fall-related physical functions, particularly in balance and gait. Intervention designers and public health practitioners can use this evidence to design targeted yoga interventions for older women. Lastly, yoga can serve as an adjunct intervention to boost exercise motivation while integrating strength, aerobic, balance, gait, and flexibility training as part of a multicomponent exercise fall prevention program.

## Figures and Tables

**Table 1:** Participants, intervention, and characteristics.

Study	Participants			Control Group	Intervention		Outcomes					PEDro Score
(Country)	N	Age ± SD	Clinical Condition		Yoga Type	Time	Indicatiors	Tools	Effect Size (Cohen’s d)		Clinical Relevance	
Widjaja et al. [27] (Thailand)	N = 22	62 ± 1	Overweight	Inactive	Thai Yoga	60 mins × 3/per week Duration: 4 weeks	Balance	FRT	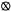	0	Trivial	7
							Gait	8UGT	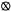	−4	Nearly Perfect	
							Gait	6MWT	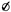	4.73	Nearly Perfect	
							LLS	CST-30	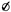	2.83	Very Large	
Widjaja et al. [27] (Thailand)	N = 22	62 ± 1	Overweight	Inactive	Thai Yoga	60 mins × 3/per week Duration: 8 weeks	Balance	FRT	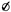	6	Nearly Perfect	7
							Gait	8UGT	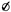	−5.69	Nearly Perfect	
							Gait	6MWT	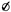	4.67	Nearly Perfect	
							LLS	CST-30	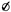	5.66	Nearly Perfect	
Marques et al. [32] (Portugal)	N = 25	83.7 ± 6.9	NA	Inactive	Chair-based Yoga	50 mins × 2–3/per week Duration: 28 weeks	Gait	2MST	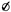	0.44	Small	7
							Gait	8UGT	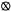	0.57	Small	
Seguin-Fowler et al. [33] (USA)	N = 38	66 ± 7.3	Chronic pain	Inactive	Flow-restorative Yoga	60 mins × 2/per week Duration: 12 weeks	Gait	8UGT	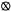	0.27	Small	6
							Gait	2MST	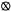	0.31	Small	
							LLS	CST-30	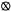	0.33	Small	
Wang. [34] (USA)	N = 18	75.5 ± 9.2	NA	Inactive	Unclear	60 mins × 2/per week Duration: 4 weeks	Balance	OLS	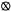	−0.12	Trivial	6
							LLS	FTSST	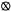	−0.41	Small	
Kuntz et al. [36] (Canada)	N = 31	65.5 ± 5.6	Knee osteoarthritis	Inactive	Biomechanically based Yoga	60 mins × 3/per week Duration: 12 weeks	Gait	40MW	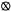	−0.47	Small	7
							Gait	6MWT	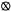	0.43	Small	
							Gait	8UGT	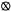	0.84	Moderate	
							LLS	CST-30	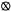	0.59	Small	
Kuntz et al. [36] (Canada)	N = 31	65.5 ± 5.6	Knee osteoarthritis	Strength training	Biomechanically based Yoga	60 mins × 3/per week Duration: 12 weeks	Gait	40MW	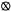	0.43	Small	7
							Gait	6MWT	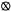	−0.33	Small	
							Gait	8UGT	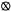	0.1	Trivial	
							LLS	CST-30	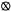	0.08	Trivial	
Cheung et al. [38] (USA)	N = 36	72	Knee osteoarthritis	Inactive	Hatha Yoga	60 mins × 1/per week Duration: 8 weeks	Balance	SPPB	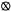	0	Trivial	6
							Gait	8UGT	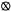	2.5	Very Large	
							LLS	FTSST	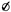	3.48	Very Large	

Footnotes: 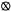 = nonsignificant between groups; 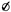 = significant between groups; LLS = lower-limb strength; FRT = functional reach test; 8UGT = 8-foot up and go test; 6MWT= 6 min walk test; CST-30 = 30 s chair stand test; OLST = one-leg-stand test; 40MW = 40 m walk; SPPB = short physical performance battery; 2MST = 2 min step test; FTSST = 5 times sit to stand test.

**Table 2:** Types of yoga used in included studies.

Type of Yoga	Description	Included Pose
Hatha yoga	Focus on breathing, meditation, and relaxation.	Mountain pose, warrior two pose, chair pose, bound angle pose, and bridge pose, tree pose, easy seated pose, open-angle pose, half locust variation, standing forward fold, reclining hamstring stretch, reclining twist, and relaxation pose.
Chair-based yoga	Focus on breathing, meditation, and relaxation.	Seated poses such as seated forward bend (Paschimottanasana), butterfly (Baddha Konasana), seated spinal twist (Ardha Matsyendrasana), and cow face pose (Gomukhasana). Gentle floor poses like cat (Cakravakasana), child’s pose (Balasana), snake (Bhujangasana), and child’s pose with arms (Utthita Balasana) provided restorative stretches. Standing poses such as side bending stretch (Tiryaka Tadasana), dorsal torsion (Kati Chakrasana), triangle (Trikonasana), and eagle or half eagle (Garudasana) challenged participants’ balance and posture.
Flow-restorative yoga	Focus more on gentle poses and supportive props, emphasize on breathing and relaxation.	Three parts breathe, seated side flow, cate/dog, leg extension, hip circles, rocking flow, lunge twist flow, mountain pose, greaser arms, bird wings, warrior I and II to peaceful warrior, boat pulse, bridge kriya, supine twist flow, legs up the wall, supported reclined bound angle, savasana.
Biomechanically based Yoga	Improving lower-extremity strength and hip mobility.	Squats, lunges, supported lunges, and bridge poses
Thai Yoga	The main exercise phase included dynamic exercises with slow, repeated movements (2 times) and static exercises where each pose was held for 15 s.	Researchers selected 28 poses from the book 127 Postures of Thai Yoga Exercise. Some poses were modified for better balance, especially for overweight/obese participants.

**Table 3:** Risk of bias assessment of the included studies.

Study	1	2	3	4	5	6	7	8	9	10	11	Overall
Widjaja et al. [27]	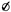	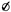	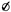	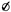	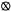	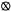	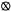	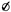	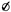	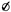	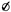	7
Marques et al. [32]	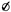	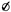	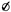	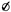	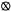	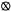	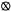	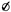	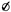	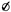	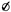	7
Seguin-Fowler et al. [33]	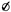	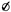	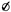	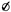	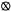	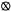	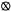	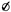	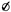	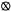	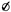	6
Wang [34]	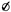	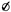	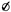	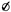	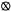	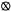	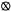	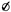	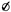	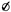	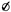	7
Kuntz et al. [36]	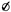	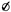	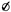	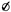	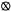	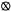	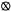	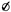	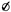	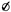	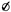	7
Cheung et al. [38]	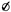	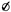	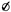	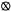	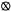	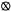	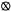	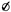	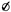	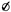	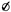	6

Footnotes: 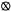 = does not meet the criteria; 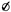 = meets the criteria.

## Appendix A

**Table A1:** The details of the search query in each database.

Database	Search Query	Results	Time
PudMed	(elder OR older OR age OR senior) AND (women OR female) AND (yoga) AND (fall OR physical OR balanc* OR mobility OR strength OR gait) **Filters: Randomized Controlled Trial**	179	1 January 2025 15:36
EBSCO	((elder OR older OR age OR senior) AND (women OR female) AND (fall OR physical OR balanc* OR mobility OR strength OR gait)) AND TI yoga **Source Types: Academic Journals** **Persistent link to search: https://reurl.cc/A63KpQ** (accessed on 1 January 2025)	316	1 January 2025 16:01
ProQuest	summary((elder OR older OR age OR senior) AND (women OR female) AND (fall OR physical OR balanc* OR mobility OR strength OR gait)) AND title(yoga) **Source Types: Academic Journals** **Filters: Language [English]**	81	1 January 2025 16:24
Scopus	TITLE (elder OR older OR age OR senior) AND (women OR female) AND TITLE (yoga) AND ABS (fall OR physical OR balanc* OR mobility OR strength OR gait) AND (LIMIT-TO (DOCTYPE, “ar”)) AND **(LIMIT-TO (LANGUAGE, “English”) OR LIMIT-TO (LANGUAGE, “Chinese”))**	110	1 January 2025 16:46

Footnotes: Bold text indicates the use of the filtering function of each electronic database.
